# Anti-Osteoarthritis Mechanism of the Nrf2 Signaling Pathway

**DOI:** 10.3390/biomedicines11123176

**Published:** 2023-11-29

**Authors:** Sarmistha Saha, Nazih Y. Rebouh

**Affiliations:** 1 Department of Biotechnology, Institute of Applied Sciences & Humanities, GLA University, Mathura 281406, Uttar Pradesh, India; 2 Department of Environmental Management, Institute of Environmental Engineering, RUDN University, 6 Miklukho-Maklaya St., 117198 Moscow, Russia

**Keywords:** Nrf2, osteoarthritis, inflammation

## Abstract

Osteoarthritis (OA) is a chronic degenerative disease and the primary pathogenic consequence of OA is inflammation, which can affect a variety of tissues including the synovial membrane, articular cartilage, and subchondral bone. The development of the intra-articular microenvironment can be significantly influenced by the shift of synovial macrophages between pro-inflammatory and anti-inflammatory phenotypes. By regulating macrophage inflammatory responses, the NF-κB signaling route is essential in the therapy of OA; whereas, the nuclear factor erythroid 2-related factor 2 (Nrf2) signaling pathway appears to manage the relationship between oxidative stress and inflammation. Additionally, it has been demonstrated that under oxidative stress and inflammation, there is a significant interaction between transcriptional pathways involving Nrf2 and NF-κB. Studying how Nrf2 signaling affects inflammation and cellular metabolism may help us understand how to treat OA by reprogramming macrophage behavior because Nrf2 signaling is thought to affect cellular metabolism. The candidates for treating OA by promoting an anti-inflammatory mechanism by activating Nrf2 are also reviewed in this paper.

## 1. Introduction

Articular cartilage deterioration is a hallmark of osteoarthritis (OA), a chronic degenerative condition [1]. Articular cartilage degeneration is a hallmark of osteoarthritis (OA), a chronic degenerative disease [2,3,4]. The World Health Organization (WHO) has classified OA as a “priority disease” since it is the primary global cause of chronic impairment in those over 70. OA is identified as the advanced stage of joint degeneration, which may be precipitated and spread by trauma to the cartilage, ligaments, or other joint tissues (post-traumatic OA), among other causes. The causes of OA can be attributed to a wide range of mechanical, metabolic, and hereditary variables [4]. The risk factors for OA are age, gender, race, occupation, obesity, hypertension, aberrant joint strength lines, weak muscle strength, and a history of joint damage [5]. An inflamed synovium, oxidative stress, apoptosis in chondrocytes, a degrading cartilage extracellular matrix, and subchondral bone sclerosis are all results of OA. New information has emerged regarding the roles of the synovial lymphatic system and programmed cell death in the pathogenesis of OA [6].

Currently, available OA therapeutic strategies include surgical intervention, pharmaceutical techniques, and nonpharmacological approaches (such as diet and exercise) [7]. Although numerous pharmacologic strategies have been vigorously pursued and some medications have shown promise in preclinical investigations, none have emerged with any major clinical success [8]. As a result, there is a critical medical need to create new interventional platforms for OA treatment that are more effective. Non-steroidal anti-inflammatory drugs (NSAIDs) are currently used as therapeutic agents for OA; however, they offer only short-term pain relief with adverse side effects and toxicity [9]. Recent research has demonstrated a direct crosslink between joint inflammation and oxidative stress with the development of OA [10].

Chondrocyte function and viability are compromised by inflammatory mediators, mechanical stress, and oxidative stress, which reprogram the cells to undergo hypertrophic differentiation and early “senescence” and make them more vulnerable to the effects of pro-inflammatory and pro-catabolic mediators. The catabolic factors produced by OA chondrocytes, which mimic terminally developed chondrocytes in the growth plate and actively create pro-inflammatory cytokines and enzymes that break down the matrix, cause additional cartilage deterioration [11]. The age-related decrease in chondrocyte function is also accompanied by progressive chondrocyte dysfunction, which is indicated by the expression of senescence-associated markers, telomere length elongation, and mitochondrial dysfunction [12]. The production of synovial fluid, which enables the joint to move freely, is the synovium’s primary function; the fluid lines the joint cavity. As OA progresses, the concentrations of synovial fluid constituents, including lubricin and hyaluronic acid, decline, affecting the cartilage integrity. The layers of the synovium lining and sublining are the two main regions that make up the synovium. The synovial lining is made up of macrophages and fibroblast-like cells. Fibroblasts, macrophages, adipose tissue cells, and blood vessels make up the synovial sublining [13]. It has been noted that synovial inflammation occurs both early and late in OA. Following that, matrix metalloproteinases (MMPs) and cytokine production (IL-1β and TNF-α) cause cartilage degradation and bone erosion over time [14,15]. The activation of transcription factors, including NF-κB and Nrf2, is a crucial part of oxidative stress responses and inflammatory signaling cascades [16]. This review summarizes data supporting the role of Nrf2 activation or inhibition in the development or treatment of OA by addressing oxidative stress and inflammation.

## 2. Correlation between Oxidative Stress and Inflammation in Osteoarthritis

An imbalance between the formation of reactive oxygen species (ROS) and the reaction of antioxidant proteins is referred to as oxidative stress. A variety of biochemical, metabolic, and genetic pathways help cells maintain this equilibrium of oxidants and antioxidants and, when it is out of balance, pathophysiological effects can result [17]. Low levels of ROS production are typical in cells and they are necessary for preserving cellular homeostasis and function [18]. But when this physiological system is out of balance, inflammatory cytokines and chemokines are expressed more frequently, which changes the function of cellular macromolecules, like proteins, lipids, and DNA, by oxidizing them. The primary locations of ROS production are mitochondria and peroxisomes, which include NADPH oxidases (NOXs), nitric oxide synthase (NOS), and xanthine oxidase [19]. The NOX complex is made up of two membrane-associated (p22phox and gp91phox) and three cytosolic (p40phox, p47phox, and p67phox) protein components [20]. The only form of cartilage cell that is a resident, the chondrocyte, expresses NOX components, which are the main ROS producers [21]. During pathological circumstances, cytosolic units migrate to the inner surface of the plasma membrane, where they attain an active enzyme complex, resulting in the production of ROS [22]. On the other hand, there are three different NOS isoforms, including neuronal NOS (nNOS), endothelial NOS (eNOS), and inducible NOS (iNOS); iNOS is activated by inflammatory cytokines, generating a high level of NO and requiring a low calcium level [23]. In order to maintain the physiological balance of the cellular redox status and to control the cellular response to stress and inflammation, it is hypothesized that the inflammation and oxidative stress work in concert with each other. Inflammatory cytokines, like interleukin-1 (IL-1), tumor necrosis factor (TNFα), interferon-γ (IFN-γ), and IL-17, among others, stimulate chondrocytes to express iNOS, which is then substantially elevated in the cells [24]. Excessive ROS act as secondary messengers and promote cartilage deterioration by upregulating the production of matrix-degrading proteases, lowering the synthesis of the extracellular matrix (ECM), and triggering apoptosis in chondrocytes. Numerous investigations have demonstrated that human OA cartilage and chondrocytes have significantly elevated ROS levels [25,26,27,28]. In OA and aged cartilage, peroxiredoxins (Prx3; mitochondrial Prx) were shown to be hyperoxidized, indicating higher oxidative stress [29]. Endogenously generated NO prevented the production of proteoglycans in the superficial and deep cartilage zones in cultures of human articular chondrocytes [30]. Also, the menadione treatment of OA chondrocytes revealed elevated levels of oxidized Prx3, which were connected to lowered Akt and elevated p38 pro-death signaling [31]. Menisci and the infrapatellar pad are examples of soft tissues that are important in the etiology of knee OA. A meniscal tear leads to knee OA; however, the meniscal structural breakdown and weakening brought on by knee OA can also result in a spontaneous meniscal tear [32]. Moreover, a new source of adipokines and inflammatory mediators that accelerates the development of knee OA is the infrapatellar fat pad [33]. As a result, further research is necessary to determine whether there is a connection between oxidative stress and infrapatellar adipose tissue in knee OA.

The antioxidant defense system consists of several enzymes, including catalases, peroxiredoxins, glutathione peroxidase, and superoxide dismutases (SODs), as well as nonenzymatic components, like glutathione (GSH), ascorbic acid, tocopherol, etc [34]. Numerous in vitro and in vivo investigations have demonstrated that chondrocytes’ upregulated cellular antioxidant defense system inhibits the production of catabolic genes. It has been shown that the survival of chondrocytes in pathological conditions depends on the autophagic clearance of defective mitochondria and reduction of ROS [25]. Mitochondrial malfunction or the dysregulation of SOD2 expression may result in excessive ROS generation, which may harm chondrocytes and cause cell death [29]. Increased oxidative stress is positively correlated with collagen degradation, pointing to a possible function for ROS in the breakdown of the cartilage matrix [26].

Inducible nitric oxide synthase (iNOS), metalloproteinase with thrombospondin 5 (ADAMTS5), MMP-3, COX-2, NO, TNF-α, interleukin-6 (IL-6), and prostaglandin-E2 (PGE2) are a few examples of catabolic and inflammatory molecules that are influenced and enhanced by IL-1β production. However, a tight-junction-mediated barrier made up of a fraction of epithelial-like CX3CR1^+^ tissue-resident macrophages may be able to control the inflammatory response [35]. Macrophages that are identified by the general surface antigens CD14 and F4/80, which are responsible for inducing the innate immune response, are the primary cause of OA [36]. Additionally, there are alternately activated anti-inflammatory phenotypes (CD163 and CD206 as M2 surface markers) and traditionally activated pro-inflammatory phenotypes (CD80, CD86, and CD11b as M1 surface markers) among the plastic diverse phenotypes of macrophages [37].

Recent research has shown that chronic unresolved inflammation, along with oxidative stress, plays a crucial part in the onset and progression of OA [38,39]. Different inflammatory mediators have been linked to the pathophysiology of OA, as observed in the tissues of both human OA patients and animal OA models [4]. Damage-associated molecular patterns (DAMPs) that bind to so-called pattern recognition receptors (PRRs) are the primary mechanism through which OA is connected with innate immune activation [40]. These fragments are thought to be the initial catalyst for the inflammatory response in the OA joint [41]. It has been demonstrated that a wide variety of extracellular matrix fragments from damaged cartilage can bind to TLR-2 and TLR-4. When conditions are not favorable, chondrocytes, which are typically dormant cells, become active and release a large number of pro-inflammatory cytokines and chemokines, which trigger collagenase expression, leading to the cartilage ECM breakdown [42]. Additionally, chondrocytes have receptors for several cytokines and chemokines that promote inflammation. Increased levels of cytokines in joints play a crucial role in OA pathology by regulating oxidative stress, cartilage ECM turnover, and chondrocyte death [43]. In a preclinical model of OA, macrophage depletion by clodronate significantly decreased the MMP-mediated degradation of articular cartilage and the development of osteophytes [44]. By increasing the expression of catabolic genes, such as COX-2, IL-6, iNOS, and collagenases, primary chondrocytes and cartilage explants stimulated with IL-1β and TNFα imitate the diseased circumstances that exist in vivo [45,46]. It has been reported that ADAMTS5 has much higher aggrecanolytic activity compared with human ADAMTS4 [47]. ADAMTS5 is shown to be the major aggrecanase involved with OA pathogenesis using knockout mouse models [47]. As per their report, compared to ADAMTS4, the catalytic domain of ADAMTS5 has a greater intrinsic catalytic ability. When compared to normal chondrocytes, OA chondrocytes exhibit a much higher level of IL-1RI expression [48]. The intra-articular injection of TNF-α or IL-1, alone or in combination, accelerated the progression of OA in rabbit knee joints [49]. In a mouse model, the intra-articular injection of the protein IL-6 increased cartilage degradation [50]. In contrast, the severity of age-related OA in male mice in a study utilizing IL-6 knockout mice was dramatically elevated; however, it was not elevated in female mice, indicating that chondrocyte homeostasis may depend on low levels of IL-6 [51]. When chondrocytes are stimulated with IL-1β, a series of processes take place that result in the activation of p38, JNK, ERK-MAPK, and PKC, as well as the nuclear translocation of NF-κB, STATs, and ATFs [42,52,53]. The expression of IL-6, COX-2, iNOS, and its metabolites PGE2 and NO, as well as other pro-inflammatory phenotypic abnormalities in chondrocytes, is influenced by elevated ROS levels, which also activate AP1. Additionally, a higher pro-oxidant load can block PI3/Akt signaling and activate the MEK/ERK signaling pathway, which both limit proteoglycan formation [54]. In bovine chondrocytes, TNFα enhanced the expression of cFos/AP1 through the generation of ROS by NADPH oxidase [55]. The generation of ROS is necessary for IL-1β to trigger the expression of cFos and MMP-1 in chondrocytes [56]. In OA, it has been reported that small molecule inhibitors of STAT3 signaling or the antibody-mediated neutralization of systemic levels of IL-6 can reduce cartilage breakdown [57].

S100B is believed to have pro-inflammatory and pro-catabolic effects, primarily through chondrocytes’ RAGE-dependent signaling [58]. As a result, chondrocytes exhibit enhanced MMP13 expression [59]. Additionally, OA has been linked to increased S100A11 synthesis, whose chondrocyte secretion is induced by agents like TNF-α and IL-8 [60]. S100A12, a protein closely related to S100A8 and S100A9, is significantly elevated in the synovial fluid of OA patients [61]. Patients with OA have also reported high levels of S100A8/A9 in their synovial fluid and blood [62]. As evaluated by several inflammatory cell layers, S100A8/A9 has been demonstrated to cause enhanced synovial activation [62]. A subsequent investigation supported the notion that S100A8/A9 primarily mobilizes pro-inflammatory Ly6C^high^ monocytes toward the inflamed synovium [63]. In fact, the S100A9 activation of human OA synovial tissue and macrophages causes a rise in pro-inflammatory and catabolic factors via TLR-4 signaling [64]. Interestingly, S100A8/A9 also triggers the anabolic process of ectopic bone formation and activates Wnt signaling to promote bone formation [65].

## 3. Role of the Nrf2 Signaling Pathway in Osteoarthritis

**Figure 1:**
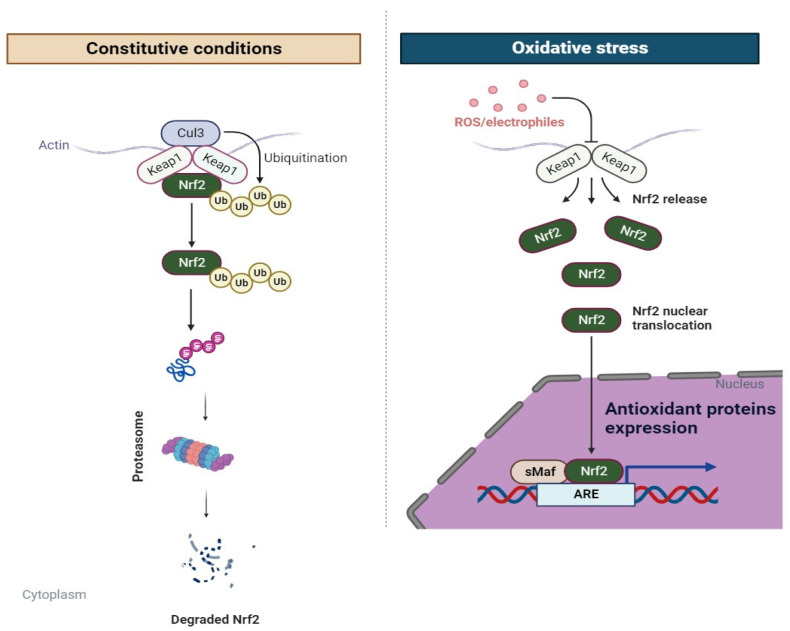
Overview of the Nrf2 signaling pathway.

Among the key systems, Nrf2/Keap1 signaling systems play a regulatory role in the crosslink of antioxidant and inflammatory systems [16]. In order to maintain the redox state and the production of antioxidant genes, Nrf2 and its antagonistic regulator, the E3 ligase adaptor Kelch-like ECH-associated protein 1 (Keap1), are essential [16]. A transcription factor known as Nrf2 (NF-E2-related factor 2) is a member of the Cap’n’collar (CNC) family of bZIP transcription factors [66] (Figure 1). Another protein of interest is Keap1, with 624 amino acids and 27 cysteine residues [66]. Keap1 is composed of five domains: a C-terminal domain (CTR), an N-terminal region (NTR), a tramtrack and bric-à-brac domain, a central intervening region (IVR) with six kelch repeats, and a nuclear export signal that regulates Keap1’s cytoplasmic localization [67]. While Keap1, Nrf2’s antagonist, controls the N-terminal domain’s stability and ubiquitination, the Neh5 domain controls Nrf2’s cytoplasmic localization [68]. The nuclear localization signal (NLS), which is in charge of the nuclear translocation of Nrf2, and the basic-region leucine zipper (bZIP) domain, which controls DNA binding, are both present in the Neh1 domain [69]. The transactivation domains Neh3, Neh4, and Neh5 mediate the interaction of Nrf2 with other coactivators [70]. A negative regulatory domain called Neh6 with serine-rich residues interacts with a protein called β-TrCP that contains β-transducin repeats, which causes Nrf2 to be ubiquitinated [71]. The Neh7 domain stimulates the binding of Nrf2 to retinoic X receptor α (RXRα), which suppresses the Nrf2–ARE signaling pathway [72]. The Neh2 domain has two peptide binding motifs (ETGE and DLG) that let Nrf2 bind to different proteins, which regulates Nrf2 stability, and seven lysine residues that affect ubiquitin conjugation [16]. Under normal circumstances, Keap1, a substrate adaptor protein for the Cullin-3-dependent E3 ubiquitin ligase complex that promotes Nrf2 ubiquitination and its proteasomal destruction, interacts with the ETGE and DLG motifs [73]. However, the E3-ligase complex undergoes a conformational shift under oxidative stress, preventing Nrf2 from interacting with the ubiquitin-conjugating mechanism [66]. As a result, the Nrf2 is liberated from the complex, moving into the nucleus where it forms a heterodimer with the sMaf protein before being activated by the ARE, which further controls the production of antioxidant proteins and cell defense systems [66]. The Nrf2 pathway stimulates the expression of genes linked to NADH regeneration and redox detoxification [74]. To prevent oxidative stress-related cell damage and preserve redox equilibrium, heme oxygenase-1 (HO-1), glutathione S-transferase (GST), and reactive oxygen species (ROS) elimination mechanisms are finally translated and activated [75]. Numerous oxidative stress-related diseases, such as neurodegenerative disorders, cardiovascular abnormalities, and pulmonary disorders, are linked to Nrf2 signaling [76,77,78]. Also, an anti-inflammatory phenotype is produced by macrophages’ altered redox state and metabolism as a result of Nrf2 transcriptional activation [79].

Nrf2 activators are, therefore, thought of as possible tools for increasing antioxidant capacity and treating disease. Several plant-based natural products, including curcumin, sulforaphane, and resveratrol, have been developed as Nrf2 activators while several activators are being tested in clinical settings as potential therapeutics [80]. However, there are some challenges that also exist [81]. The majority of Nrf2 activators are electrophilic and quickly metabolized, which may result in limited bioavailability in distant organs, which is connected to low effective biological concentration [82]. Also, some Nrf2 activators may affect other signaling pathways and interfere with relevant biological processes in addition to activating Nrf2 and triggering antioxidant enzymes [83].

## 4. Crosstalk of Nrf2 and NF-κB Signaling in Osteoarthritis

**Figure 2:**
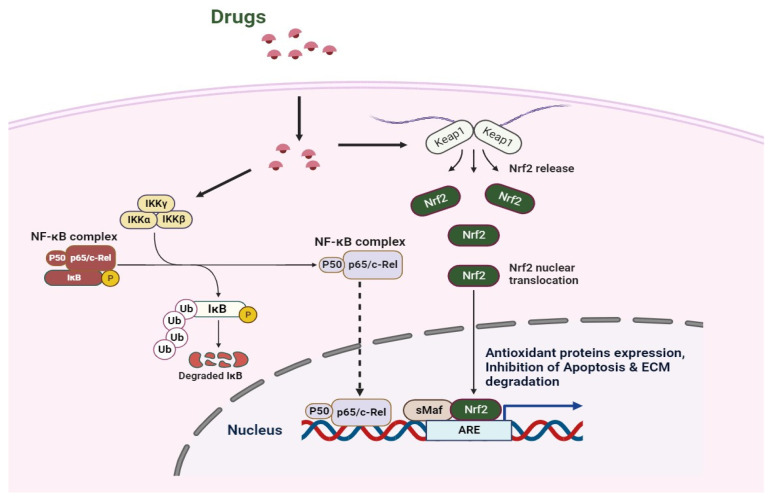
Schematic overview of the mechanism involved in the chondrocytes when treated by the drugs.

Nrf2 activation has been found to functionally couple the suppression of NF-κB transcriptional activity, hence regulating redox homeostasis and inflammatory responses [16,84] (Figure 2). On the one hand, HO-1 can prevent IκB-α from degenerating, which stabilizes NF-κB in the cytoplasm [85]. Conversely, CO produced by HO-1 can bind to downstream transcription factor IRF3 and disrupt the downstream pathway, hence inhibiting TLR/NF-κB signaling [86]. Notably, new anti-inflammatory medications can reduce inflammation by blocking NF-κB and turning on Nrf2 [87]. Additionally, by regulating the production of detoxifying enzymes to buffer reactive oxygen species, Nrf2 activation can influence the antioxidant response. Thus, the reduction in ROS leads to the suppression of oxidative stress-mediated NF-κB activation [88]. Nrf2 suppresses oxidative stress-mediated activation by lowering ROS levels within cells, which results in an anti-inflammatory M2 phenotype. Furthermore, Nrf2 can adversely influence NLRP3 inflammasome activity because NLRP3 inflammasome signaling is another NF-κB activator [89]. Comparably, Nrf2 can raise NQO1 synthesis and have a detrimental impact on the activation of the NLRP3 inflammasome [89]. On the one hand, IκB-α functions as the primary inhibitor of NF-κB, which is capable of being released into the pro-inflammatory microenvironment when it is phosphorylated by IκB kinase (IKK) β. IKKβ can mediate the degradation of ubiquitin and proteasome by binding KEAP1 through the ETGE motif. KEAP1 can be blocked when IKKβ is stabilized; whereas, NF-κB can be activated and Nrf2 inhibited when IκB-α is phosphorylated [90]. Conversely, KEAP1 has been shown to be a partner of the NF-κB p65 subunit and their contact can block the Nrf2–ARE pathway [91]. Therefore, the NF-κB-induced production of pro-inflammatory cytokines can be inhibited by Nrf2 activation [92]. Moreover, it has been found that treating OA involves M2 polarization brought on by Nrf2 activation [93].

## 5. Potential Therapeutic Agents of Osteoarthritis via the Nrf2 Signaling Pathway

Despite significant efforts over the past few decades to translate preclinical results into clinical practice, the majority of clinical trials utilizing general antioxidant or anti-inflammatory therapy have failed, most likely as a result of a lack of understanding of the signaling pathways in health and disease. A logical strategy may be devised to enhance therapeutic intervention via a better knowledge of the processes by which the Nrf2 signaling pathway functions, as well as their limitations and promises.

In OA, calmodulin-dependent protein kinase II (CaMKII) was phosphorylated by transient receptor potential vanilloid 1 (TRPV1) to prevent M1 macrophage polarization; however, the particular inhibitor of Nrf2 reversed the anti-inflammatory effect [94]. Additionally, the 4-octyl itaconate-induced transcription of Nrf2 in chondrocytes leads to high expressions of HO-1, NQO1, and GCLC and low secretions of IL-6, IL-10, MCP-1, and TNF-α. These changes can switch off the prevention of the cell death and apoptosis of chondrocytes by reducing oxidative stress and inflammation responses and slowing the progression of OA [95]. In the same way, the TNF-α-induced degradation of COL2 in OA can be prevented by dimethyl fumarate (DMF), which also inhibits MMP-1, MMP-3, and MMP-13 synthesis [96]. Dimethyl fumarate (DMF), is the only FDA-approved drug to treat relapsing-remitting multiple sclerosis as a potent Nrf2 activator [97]. Repeated administration of dimethyl fumarate reportedly reversed the pain-related behaviors in OA rats [98]. Trigonelline, a Nrf2 inhibitor, hindered the therapeutic action of dimethyl fumarate. Dimethyl fumarate oral treatment also improved Nrf2 expression and restored mitochondrial biogenesis. One of the study’s limitations was that they used only male rats and no female rodents were included. Dimethyl fumarate is not a specific Nrf2 agonist; thus, it could be more appropriate to utilize a unique Nrf2 agonist and transgenic mouse models to examine the development of OA pain and mitochondrial biogenesis. This was one of the study’s limitations. Additionally, monomethyl fumarate, which is produced by the metabolism of dimethyl fumarate, may have an impact on the way that pain develops in the brain. This subject should be investigated in future research following oral delivery of dimethyl fumarate.

In IL-1β-treated chondrocytes, rhoifolin, a flavonoid, reduced the expression of senescence-associated secretory phenotype (SASP) factors and the senescence phenotype [99]. Rhoifolin inhibited the NF-κB pathway’s cascade activation caused by IL-1β and knockdown studies showed that rhoifolin may bind to Nrf2 to inhibit the NF-κB pathway [99]. In another study, Notoginsenoside R1 (NR1), a novel saponin derived from *Panax notoginseng,* a well-known antioxidant, reportedly downregulates the expression of matrix-degrading enzymes, which was also accompanied by the reduction of the degradation of the extracellular matrix (ECM) in IL-1β-induced OA [100]. Additionally, following NR1 administration, the Nrf2/HO-1 signaling pathway was activated, which further downregulated the IL-1β-induced activation of the NF-κB pathway and also reduced the cartilage degeneration and OA scores of the knee. Both of these studies showed positive results; however, only the rat model was used to evaluate their preventive impacts against OA; OA patients were not included. Therefore, more clinical studies are required to determine how these molecules affect OA. Second, according to published research, these compounds utilized for molecular docking were those that regulated the NF-κB pathway only. Thus, additional research on a novel chemical that controls the NF-κB and Nrf2 pathway and has a higher affinity is warranted. Also, as these findings are based solely on theoretical calculations and animal studies, more clinical research is required before these compounds may be used to treat OA in humans.

Curcumin, a major bioactive component in turmeric (*Curcuma longa*), reportedly showed cartilage protective effects via the ROS/Nrf2/HO-1-SOD2-NQO-1-GCLC signaling axis in temporomandibular joint osteoarthritis [101]. In response to IL-1β treatment, curcumin administration raised the mRNA levels of the cartilage anabolic factors while inhibiting the production of the matrix-degrading proteinases, MMP-1, MMP-3, MMP-9, and MMP-13, inflammatory mediators, such as IL-6, iNOS, and COX-2, and oxidative stress. Similar to curcumin, quercetin can lower NO, TNF-α, and IL-1β by decreasing the NLRP3 signaling pathway, p38 activation, and endoplasmic reticulum stress [102,103]. Additionally, the activated SIRT1/AMPK signaling pathway can mediate the reversal of mitochondrial dysfunctions and the removal of ROS in chondrocytes [103,104]. Also, the administration of quercetin has been observed to upregulate TGF-β1 and TGF-β2 levels in the synovium due to an increase in M2 macrophages [104].

Morin, a natural flavonoid, activated the Nrf2 signaling pathway to reduce IL-1β-induced inflammation in human chondrocytes [105]. The outcomes showed that IL-1β greatly elevated NO, PGE2, MMP1, MMP3, and MMP13 production. Furthermore, morin therapy considerably reduced the elevation. Additionally, morin triggers the expression of Nrf2 and HO-1 with the suppression of NF-κB activation; however, Nrf2 knockdown prevented morin’s anti-inflammatory effects.

Astaxanthin, known as a “marine carotenoid”, suppresses the IL-1β-induced upregulation of iNOS and COX-2 proteins, as well as phosphorylated ERK and JNK [106]. Additionally, the MEK inhibitor PD0325901 administration significantly reduced the phosphorylation of ERK, attenuated the IL-1-induced expression of COX2 and MMP3, and reduced the expression of collagen II, indicating the involvement of the ERK/MAPK signaling pathway. Numerous studies have shown that accelerated chondrocyte apoptosis is associated with aging and the development of OA as chondrocyte reduction worsens cartilage degradation because it fails to preserve cartilage structure [107]. The findings of this study also suggest that astaxanthin can inhibit NF-κB to lessen TNF-α-induced ECM breakdown and chondrocyte death. Additionally, astaxanthin inhibits inflammation, oxidative stress, and apoptosis in mouse OA chondrocytes through activating Nrf2.

IL-1β cooperates with Th17 and NK22 cells to attract neutrophils into the tissue, which aids in the promotion of OA [108]. Carnosine (CA), is a dipeptide that contains β-alanine and L-histidine [109]. The anterior cruciate ligament transection combined with the medial meniscectomy approach is reportedly used to induce OA; whereas, in vitro studies were conducted in fibroblast-like synoviocyte (FLS) cells. Treatment with CA enhanced synovial defense and slowed cartilage deterioration while reducing zonal depth lesions. Furthermore, the CA-treated group increased the expression of COX-2 while decreasing the expression of HO-1 and Nrf2. CA increased the permeability of the mitochondrial membrane and decreased ROS levels in FLS cells.

N-acetyl-5-methoxytryptamine, also known as Melatonin, protects the chondrocytes from apoptosis, promotes anabolic metabolism, and, finally, reduces the catabolic metabolism in cartilage with redox balance [110]. The most recent research also shows that the interaction of Mel and MT1 receptors can activate PI3K/Akt and ERK signaling pathways in synovial fibroblasts, which results in the up-regulation of microRNA-185a, which can reverse OA-induced pathological consequences by lowering the secretion of TNF-α, IL-8, and vascular endothelial growth factors [111].

Akebia Saponin D, well known as Asperosaponin VI, prevents the generation of inflammatory mediators, such as COX-2, iNOS, NO, PGE2, IL-6, and TNF-α, in chondrocytes treated with IL-1β [112]. Additionally, it might encourage the formation of Aggrecan and collagen II while suppressing the development of ADAMTS5 and MMP13. The mechanistic investigation proved that ASD has an anti-inflammatory impact via activating the Nrf2 target, increasing HO-1 expression, and blocking P65 from binding to DNA.

In another study, Phillygenin, a bioactive component from the *Forsythiae Fructus*, prevented IL-1β-induced ECM breakdown and pro-inflammatory cytokine production in primary murine chondrocytes via activating Nrf2 and inhibiting the NF-κB pathway [113]. Caffeic acid phenethyl ester (CAPE), a natural flavonoid compound, reportedly protects cartilage and attenuates OA [114]. When chondrocytes were stimulated by IL1β, CAPE decreased the production of iNOS and COX-2, as well as the extracellular release of NO and prostaglandin E2 in the cell culture supernatants. Additionally, CAPE slowed down the breakdown of the ECM by upregulating aggrecan and collagen II expression while downregulating MMP3, MMP13, a disintegrin, and metalloproteinase with thrombospondin motif expression 5. Also, CAPE reduced NF-κB signaling and turned on the Nrf2 expression. In animal models, CAPE administration slowed the development of OA and prevented cartilage degeneration. According to Elmali et al.’s study [115], CAPE reduced the in vivo cartilage degradation caused by unilateral anterior cruciate ligament transection. However, the authors should have used co-immunoprecipitation; the binding between CAPE and the Keap1/Nrf2 complex needs to be verified. Furthermore, OA chondrocytes were not used as a positive control in these investigations; instead, the morphology of normal chondrocytes was the only aspect examined. Moreover, Nrf2-knockout mice must be used to assess the effects of CAPE. As a result, more research is needed to validate and clarify these findings.

Licochalcone A (Lico A), isolated from Glycyrrhiza species, prevented the LPS-induced chondrocyte pyroptosis by reducing the expression of NLRP3, apoptosis-associated speck-like protein (ASC), Gasdermin D (GSDMD), caspase-1, IL-1β, and IL-18 [116]. Lico A reportedly inhibits NLRP3 inflammasome via the Nrf2/HO-1/NF-κB axis. In mouse OA chondrocytes, Lico A’s anti-pyroptosis actions were reversible by the Nrf2 small interfering RNA (siRNA). In prior work, Lico A could inhibit Nrf2 to attenuate IL-1-induced chondrocyte inflammation [117]. Similarly, sesamin, isolated from sesame, greatly reduced the generation of PGE2 and NO brought on by IL-1β-induced OA [118]. In IL-1β-stimulated chondrocytes, sesamin reduced MMP1, MMP3, and MMP13 production. Sesamin also inhibited the IL-1β-induced phosphorylation of NF-κB p65 and IκBα. Meanwhile, sesamin was found to elevate the expression of Nrf2 and HO-1. Sesamin’s anti-inflammatory properties were, however, reversed by Nrf2 siRNA, confirming their protective activity via the Nrf2 signaling pathway. In another study, pterostilbene, an analog of phytoalexin and resveratrol, reduced the levels of COX-2, iNOS, PGE2, and NO and intracellular ROS generation, which was somewhat counteracted by Nrf2 silencing in IL-1β-induced chondrocytes [119]. Pterostilbene promoted the nuclear translocation of Nrf2 in cartilage and avoided cartilage degradation. Lutein, a tetraterpenoid, provides considerable cytoprotection against monosodium iodoacetate-induced oxidative stress, inflammation, and apoptosis in the OA model via NF-κB and Nrf2 activation [120]. S-allylmercaptocysteine (SAMC) could prevent type II collagen breakdown in the OA rats by reducing the production of metalloproteinases (MMPs) and stabilizing the extracellular matrix (ECM) [121]. These effects were parallel with NF-κB signaling inhibition and Nrf2 activation. In the SAMC-treated group, Nrf2 was activated along with its downstream gene, NQO1, while NOX4 expression was downregulated. As a result, the level of the lipid peroxidation byproduct 4-hydroxynonenal (4HNE) in articular cartilage was decreased. It is interesting to note that the SAMC’s protective impact on IL-1β-stimulated chondrocytes was lost following Nrf2 knockdown. In IL-1β-stimulated OA chondrocytes, wogonin, a well-known flavonoid, totally inhibited the expression and synthesis of inflammatory mediators, such as IL-6, COX-2, PGE2, iNOS, and NO [42]. Wogonin also demonstrates strong chondroprotective effects by reversing the catabolic ends of the matrix degradation signaling axis, suppressing the expression, synthesis, and activities of matrix-degrading proteases in osteoarthritic chondrocytes and preventing the release of s-GAG and COL2A1 in OA cartilage explants stimulated by IL-1β. A drawback of this investigation was that wogonin may not have a solely protective effect on chondrocytes; upon absorption, the molecule may undergo glucuronidation, sulfation, methylation, conjugation, and glycosylation, leading to the generation of several metabolites. Thus, all these studies that studied natural products have not considered the possible interactions of their metabolites in their chondro- and cartilage-protective activities. In a recent study in mouse OA chondrocytes, stevioside, a naturally diterpenoid glycoside, inhibited the expression of MMP-13, iNOS, COX-2, and ADAMTS4 by blocking the Nrf2/NF-κB signaling pathway [122]. This inflammation is caused by IL-1β activation. Also, the mouse DMM model’s development was considerably halted by stevioside administration. However, further research is necessary to determine whether stevioside has an anti-inflammatory effect on OA. Moreover, the relationship between the synovium and the articular cartilage and the effects on them by stevioside should also be studied.

These studies showed pharmacological benefits working against OA; however, the expression of Nrf2 may be negatively impacted by a number of issues, including target selectivity, pharmacodynamic responses, short- and long-term safety concerns, and the volatility of Nrf2 activity [123]. Nevertheless, Nrf2 is not Keap1’s sole binding partner. Additionally, p62 is expressed when Keap1 is inhibited, which may enhance Nrf2 activation. Further research is still required to fully understand the biological ramifications. Over-activation of the Nrf2/ARE signaling pathway has a risk of exacerbating the pathological OA alterations.

Another report showed cartilage destruction and subchondral bone sclerosis in the human total knee replacement samples [124]. NLRP3, ASC, Nrf2, and HO-1 were also shown to have significantly higher expression levels in the synovial tissue of OA patients. As anticipated, following Nrf2 silencing in SW982 cells, the expression of NLRP3 was increased and that of IL-1β and IL-18 was downregulated. Moreover, Nrf2 expression remained unaffected by NLRP3 knockdown. Another study revealed that kartogenin inhibited the expression of matrix degradation enzymes (MMP13 and ADAMTS5) in human chondrocytes [125]. Also, kartogenin increased the formation of the cartilage matrix in human chondrocytes treated with IL-1β. Kartogenin activated the miR-146a/Nrf2 axis to produce protective actions. These results showed that kartogenin could be able to stop OA-induced aberrant subchondral bone production; although, more research is needed to determine the underlying mechanisms. Future research should be focussed on how the kartogenin-mediated miR-146a-Nrf2 axis contributes to the degradation of the subchondral bone and articular cartilage in the advanced stages of osteoarthritis. Cheleschi et al. [126] showed that miR-146a-mediated oxidative stress generated by hydrogen peroxide elevated the expression of Nrf2 and related antioxidant enzymes in human OA chondrocytes.

A family of endogenous non-coding RNAs with a length of 18–25 nucleotides is known as a collection of microRNAs (miRNAs). In addition to being able to precisely bind to the 3′-untranslated region (3′-UTR) of the target mRNA, miRNAs also have a role in controlling the proliferation, invasion, metastasis, and death of cells [127]. Some researchers have underlined how miRNAs control inflammatory transmitters, nerve growth factors, and vascular endothelial growth factors (VEGFs), which results in their involvement in OA [128]. In addition, miRNAs can promote or reduce the expression of matrix metalloproteinase and collagen, which leads to the breakdown of the extracellular matrix and apoptosis of chondrocytes and, ultimately, OA [129]. Miyaki et al.’s research [130] identified specific miR-140 expression in cartilage tissues and its relationship to the development and homeostasis of articular cartilage; suppression of the miRNA resulted in lesions with features of age-related OA. In a recent study, the team found that IL-1-stimulated chondrocytes and OA cartilage tissues had significant levels of miR-182-5p expression [131]. The Nrf2 signaling pathway was activated in chondrocytes by miR-182-5p knockdown, which also boosted cell proliferation while reducing oxidative stress and inflammation. Some researchers assert that the expression of silent information regulation of transcription 1 (*SIRT1*) controls bone homeostasis; inhibits the advancement of OA; and promotes ECM formation, cell survival, and anti-inflammation in human OA cartilage [132]. Additionally, OA tissues and chondrocytes showed low SIRT1 expression and there was a negative correlation between SIRT1 expression and miR-182-5p expression. In the CHON-001 cells, miR-182-5p knockdown and Nrf2 pathway activation had protective benefits that were offset by SIRT1 knockdown.

The heterogeneous stromal cells known as mesenchymal stem cells (MSCs) are commonly obtained from adipose tissue, bone marrow, and umbilical cord blood. MSCs can differentiate into adipocytes, chondrocytes, and osteoblasts. In OA, several studies have revealed the potential effects of MSCs on regulating intra-articular inflammation via M2 macrophage polarization. In OA, MSCs that have been marked with iron oxide nanoparticles can cause an increase in CD206-positive cells from F4/80-positive macrophages while also causing a decrease in iNOS-positive cells from F4/80-positive macrophages [133]. Extracellular vesicles produced by MSCs from adipose tissue that have antioxidative properties as a result of overexpressing Nrf2 have anti-inflammatory and antioxidant properties. These extracellular vesicles can result in higher M2 macrophage levels as well as lower IL-6 and TNF-α levels [134].

## 6. Conclusions

The Nrf2–Keap1 signaling pathway is essential for redox signaling, regulated inflammation, and cell metabolic adaptability. In this paper, we outlined how NF-κB, Nrf2, and their interaction affect macrophage polarization and discussed the importance of macrophage phenotypes in causing and alleviating inflammation caused by OA. Studying how Nrf2 activation affects macrophages in the OA microenvironment may also point to a possible target for anti-inflammatory therapy. However, further research is still needed on a few pathways, such as how Nrf2 and JAK/STAT signaling interact in the progression of OA. It remains difficult to improve the targeting of these molecules and their activity against these multifactorial complicated disorders, despite the fact that several pharmaceutical companies are currently focusing on Keap1, the main regulator of Nrf2. Some new medications have entered clinical trials as a result of research into effective therapeutic agents that support Nrf2 activation and will likely progress the treatment of inflammation in OA. Therefore, it is conceivable to hypothesize that inhibiting Nrf2 signaling may be an effective and desirable treatment approach to avert OA.

## Data Availability

Not applicable.
